# The Nicotinic Agonist Cytisine: The Role of the NH···N Interaction

**DOI:** 10.1021/acs.jpclett.2c02021

**Published:** 2022-10-20

**Authors:** Raúl Aguado, Santiago Mata, Miguel Sanz-Novo, Elena R. Alonso, Iker León, José L. Alonso

**Affiliations:** † Grupo de Espectroscopía Molecular (GEM), Edificio Quifima, Área de Química-Física, Laboratorios de Espectroscopía y Bioespectroscopía, Parque Científico UVa, Unidad Asociada CSIC, Universidad de Valladolid, 47011 Valladolid, Spain

## Abstract

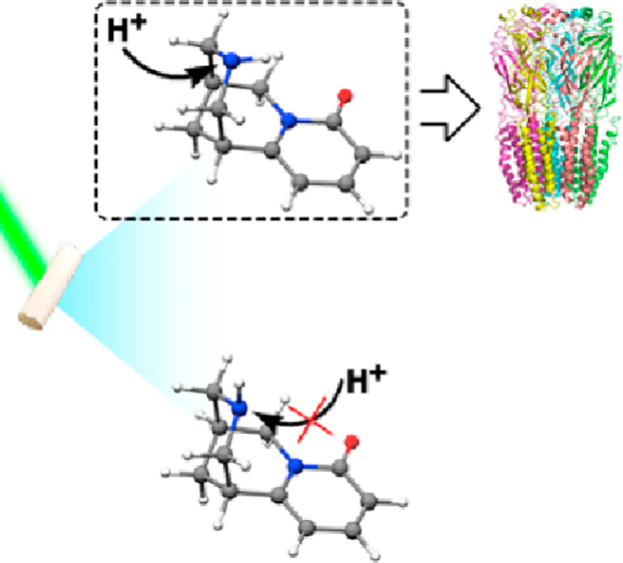

We report a detailed structural study of cytisine, an alkaloid used to help with smoking cessation, looking forward to unveiling its role as a nicotinic agonist. High-resolution rotational spectroscopy has allowed us to characterize two different conformers exhibiting axial and equatorial arrangements of the piperidinic NH group. Unexpectedly, the axial form has been found as the predominant configuration, in contrast to that observed for related molecules, such as piperidine. This anomalous behavior has been justified in terms of an intramolecular NH···N hydrogen bond. Moreover, this interaction justifies the overstabilization of the axial conformer over the equatorial one and is crucial for the mechanism of action of cytisine over the nicotinic receptor, further rationalizing its behavior as a nicotinic agonist.

The brain’s chemistry is strongly controlled by receptor–ligand interactions, a major class of intramolecular assemblies that are at the base of all biological events in living cells. In this process, an endogenous—or even an exogenous—molecule can act as a ligand for a specific receptor that receives the chemical signal and triggers the corresponding biological response.^1^ In this context, nicotinic acetylcholine receptors (NAChRs) are a well-studied family of ligand-gated ion channels that open an ion channel when activated by their specific ligand.^2^ This positive action is naturally triggered by the endogenous ligand acetylcholine, but it can also be triggered by the ubiquitous molecule nicotine. In addition, cytisine, also known as cytisinicline or sophorine, is a natural alkaloid that can produce the same response as nicotine in human neurons, acting as a nicotinic agonist.^3^ Several biomedical studies have suggested cytisine as a potent treatment to help with smoking cessation^4−6^ as it has shown superior effectiveness to nicotine^7^ and is similar to varenicline but offers lower side effects.^8^ Thus, to activate NAChRs, an exogenous molecule must be similar in shape, size, and functionalities to acetylcholine. If these conditions are fulfilled, the molecule will be capable of reaching the receptor’s active site, further triggering its biological function.^9−11^

Nicotine has been investigated in condensed phases by X-ray diffraction techniques, obtaining a single trans-configuration.^12,13^ These studies attributed the biological activity of this alkaloid to the existence and relative disposition of two key centers labeled A and B. First, a cationic center (A) is protonated under physiological conditions emulating the quaternary amine in acetylcholine. The second center (B), must be an electronegative atom that acts as a hydrogen bond acceptor. The distances between the A and B atoms range from 4.4 to 5.0 Å.^12^ Nicotine binding to NAChRs has been investigated using X-ray diffraction techniques,^14^ showing that these two centers play a crucial role in activating the nicotinic receptor. More recently, a microwave study of nicotine in the isolation conditions of a supersonic expansion^15^ revealed the existence of two trans configurations, both satisfying the two-center model.

Regarding cytisine, how can we explain its behavior as a nicotinic agonist? The answer should lie in the structural resemblance between both molecules. Based on an X-ray crystal study,^13^ this alkaloid presents three merged cycles: two chair piperidine rings (I and II) and a third saturated piperidone that confers cytisine a significant rigidity. Following the proposed two-center model, it could be inferred that the cationic center (A) might be the piperidine nitrogen, while the carbonyl oxygen can be ascribed to the B center. However, in contrast to nicotine, cytisine presents axial or equatorial arrangements of the piperidine amino (N_I_–H) group (see Figure 1b), which X-ray techniques can not discriminate. This arrangement plays a crucial role in modulating cytisine’s biological behavior in the human body since the axial form offers the most favorable position for a proton attack in activating cytisine to bind the receptor.^16^ If cytisine behaves as piperidine, where the equatorial form is the dominant one, it will not fully explain the role of cytisine as a nicotinic agonist.

**Figure 1:**
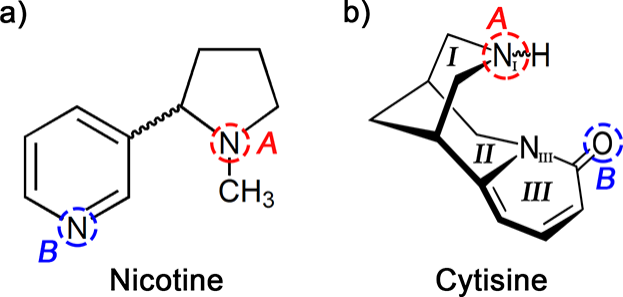
(a) Schematic structure of nicotine. The cationic (protonated) center (A) and the electronegative center (B) are depicted. (b) Sketch of the structure of cytisine highlighting the suggested A and B centers. The A center can exhibit axial and equatorial arrangements arising from the different configurations of the piperidine ring (I).

To unravel cytisine’s axial/equatorial equilibrium ratio and its role as a nicotinic agonist, it is therefore mandatory to investigate its structure using a high-resolution spectroscopic technique. Microwave spectroscopy has proven to be the only one capable of reaching such a wealth of detail,^17^ thus discriminating between cytisine’s axial and equatorial configurations as reported for piperidine.^18^ Cytisine is a solid with a high melting point (mp 156 °C) and low vapor pressure, preventing its transfer to the gas phase to perform a rotational study using conventional heating methods. To overcome this problem, our group has developed Fourier-transform microwave techniques coupled to laser ablation devices,^19^ used to reveal the unbiased gas-phase structure of relevant systems [see refs (20−23) and references therein]. We have vaporized solid cytisine, recorded its broadband spectrum in the 3.0 to 14.0 GHz region (see Figure 2a and Figure S3), and faced the spectrum analysis. We have modeled the axial and equatorial conformers by DFT computations (see Supporting Information).^24^ Using the predicted spectroscopic parameters collected in the first section of Table 1 to guide our spectral search. We anticipate that the recorded lines should present a ^14^N hyperfine structure arising from the nuclear quadrupole coupling interaction generated by the two ^14^N_I_ and ^14^N_III_ nuclei of cytisine with a nonzero quadrupole moment (*I* = 1). They interact with the electric field gradient created by the rest of the molecule, leading to a very complex hyperfine pattern for each rotational transition.^25−27^

**Figure 2:**
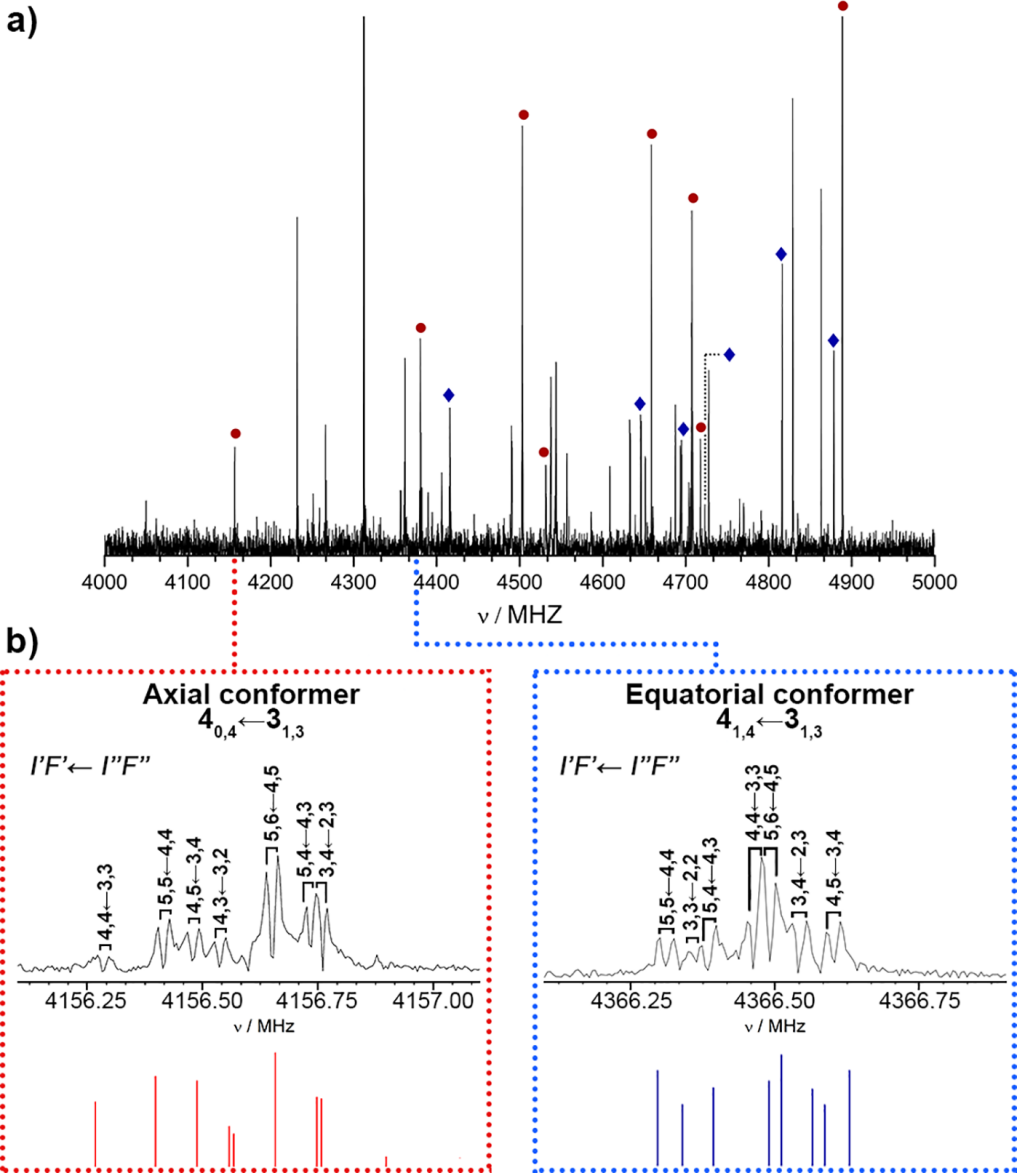
(a) Section of the broadband LA-CP-FTMW spectrum from 4 to 5 GHz. Transitions assigned to rotamer I are labeled in red, while transitions assigned to rotamer II are marked in blue; (b) Completely resolved hyperfine structure for the 4_0,4_ ← 3_1,3_ and 4_1,4_ ← 3_1,3_ rotational transitions belonging to the axial and equatorial conformers, respectively, using the LA-MB-FTMW spectrometer. Each transition appears as a Doppler doublet and the resonance frequency is determined by the arithmetic mean of two Doppler components. The energy levels are labeled with the quantum numbers *K*_*a*_, *K*_*c*_, *I*, and *F* and the quadrupole coupling Hamiltonian was set up in the coupled basis set (*I*_1_, *I*_2_, *I, J, K*, and *F*), where *I*_1_ + *I*_2_ = *I*, and *I + J = F*. The corresponding predicted spectra are also included at the bottom for comparison.

**Table 1:** Theoretical Prediction and Experimental Spectroscopic Parameters for Both Observed Rotamers of Cytisine

	theoretical B3LYP-GD3/aug-cc-pVTZ		LA-CP-FTMW spectroscopy		LA-MB-FTMW spectroscopy	
parameter	axial	equatorial	rotamer I	rotamer II	rotamer I	rotamer II
*A*^a^	1241.4	1253.1	1237.5720(33)^i^	1249.5815(98)	1237.5704(15)^i^	1249.6593(19)
*B*	647.3	645.2	648.9721(15)	647.9141(33)	648.9732(10)	647.9137(39)
*C*	518.8	515.8	519.42718(78)	517.3335(10)	519.42978(55)	517.3346(10)
|μ_*a*_|^b^	2.8	4.6	yes	yes	yes	yes
|μ_*b*_|	2.9	3.3	yes	yes	yes	yes
|μ_*c*_|	1.6	0.6	yes	no	yes	no
χ_*aa*_ (N_III_)^c^	0.9170	0.989	–	–	0.949(25)	0.894(37)
χ_*bb*_ (N_III_)	1.5594	1.563	–	–	1.570(29)	1.620(41)
χ_*cc*_ (N_III_)	–2.4764	–2.552	–	–	–2.519(29)	–2.514(41)
χ_*aa*_ (N_I_)	–1.2872	–4.937	–	–	–1.023(14)	–4.632(49)
χ_*bb*_ (N_I_)	2.7780	2.618	–	–	2.606(19)	2.568(89)
χ_*cc*_ (N_I_)	–1.4908	2.319	–	–	–1.583(19)	2.064(89)
σ_rms_^d^	–	–	41.5	39.8	1.5	2.9
*N*^e^	–	–	100	56	17	20
Δ*E*^f^	0.00	179	–	–	–	–
Δ*E*_*ZPE*_^g^	0.00	152	–	–	–	–
Δ*G*^h^	0.00	161	–	–	–	–

a *A*, *B*, and *C* are the rotational constants (in MHz).

b |μ_*a*_*|*, |μ_*b*_|, and |μ_*c*_| are the absolute values of the dipole moment (in debyes).

c χ_aa_/χ_bb_/χ_cc_ are the ^14^N nuclear quadrupole coupling constants (in MHz).

d σ_rms_ is the root-mean-square deviation of the fit (in kHz).

e *N* is the number of measured frequency centers (in the CP technique) or hyperfine components (in the MB technique) included in the fit.

f Δ*E* are energies relative to the global minimum.

g Δ*E*_ZPE_ are energies relative to the global minimum taking into account the zero-point energy (ZPE).

h Gibbs energies relative to the global minimum calculated at 298 K (all energies are expressed in cm^–1^).

i The numbers in parentheses are the 1σ uncertainties in units of the last decimal digit.

We first removed known lines belonging to photofragmentation products and managed to identify an intense set of μ_*a*_-type *R*-branch transitions of a first rotamer. The analysis was completed by predictions and measurements of other μ_*b*_- and μ_*c*_-type transitions. We discarded the rotational transitions of rotamer I from the spectrum and analyzed the remaining lines looking for a second rotamer. Hence, a weaker progression of μ_*a*_- and μ_*b*_-type *R*-branch transitions was easily identified. As mentioned earlier, most transitions appeared to be broadened by the ^14^N hyperfine structure; our LA-CP-FTMW broadband technique does not provide enough resolution to resolve them thoroughly. Thus, the frequency centers of 100 and 56 transitions measured for rotamers I and II were submitted separately to a rigid rotor analysis, which provided an initial set of rotational constants, collected in the second section of Table 1.

A first comparison between the predicted and experimental values of the rotational constants in the first two sections of Table 1 indicates that the two detected species correspond to the axial and equatorial forms of cytisine. However, we cannot discern between them; the different orientation of the terminal N_I_–H group does not cause a significant change in the mass distribution and, consequently, in the rotational constants’ values. Additional information can be obtained from the trend in the variation of the rotational constants. The observed changes, when moving from rotamer I to II, match the predicted differences between equatorial and axial conformers (see Table 1). We can then tentatively assign rotamer I as the axial form and rotamer II as the equatorial. Further support comes from the dipole moment selection rules; the nonobservation of c-type lines for the second rotameric species suggests that rotamer II is the equatorial form, as the dipole moment along this axis is predicted to be very low.

In a quest to distinguish definitely between the two conformers, we considered a dedicated experimental approach to extract information from the ^14^N nuclear quadrupole hyperfine structure. The ^14^N_I_ and ^14^N_III_ nuclei introduce hyperfine rotational probes at defined sites of cytisine and act as a probe of the chemical environment, position, and orientation of both quadrupolar nitrogen nuclei.^28^ As the axial and equatorial forms only differ in the piperidinic amino arrangement, the characterization of the ^14^N_I_ nucleus environment is, therefore, a precious spectroscopic tool in conformational identification.^29^ With this aim, we took advantage of the sub-Doppler resolution achieved with our cavity-based LA-MB-FTMW technique^30^ to fully resolve the hyperfine structure of several transitions already assigned in the broadband spectrum (see Figure 2b). All the measured hyperfine components, listed in Tables S4 and S5, were fitted to a rigid-rotor Hamiltonian supplemented with a term to account for the nuclear-quadrupole coupling contribution.^31^ The resulting rotational and quadrupole coupling constants are presented in the third section of Table 1. The excellent matching between the theoretical and experimental values of the diagonal elements of the nuclear quadrupole coupling tensor (χ_aa_, χ_bb_, and χ_cc_) provides an irrefutable identification of equatorial and axial forms of cytisine. Note that the predicted values present a drastic change in the case of the ^14^N_I_ nucleus, directly related to the different axial and equatorial arrangements of the N_I_–H group. Thus, the experimental values of the χ_aa_ and χ_cc_ diagonal elements of the ^14^N_I_ nuclear quadrupole coupling tensor vary from −1.023(14) to −4.632(49) and from 1.583(19) to 2.064(89), respectively, in excellent agreement with the predicted values shown in Table 1.

We have estimated the relative abundances of the axial and equatorial forms by comparing the intensity of rotational transitions, correcting them by the predicted values of the dipole moment components. The results show the axial form as the dominating structure (see Figure 2a) with a 3 to 1 ratio, which is in clear disagreement with piperidine, the reference molecule.^18^ This deviation of the axial/equatorial ratio must be attributed to the existence of an exotic N_I_–H···N intramolecular hydrogen bond over stabilizing the axial form. To further understand the role of this interaction, we performed noncovalent interactions (NCI) computations^32^ and a complementary Natural Bonding Orbitals (NBOs) analysis^33^ (see section S2 in the Supporting Information for detailed information). The results in Figure S2 confirm that there is a moderately strong N_I_–H···N interaction in the axial conformer, which is the driving motive stabilizing this form over the equatorial one.

As mentioned above, the experimentally observed predominance of the axial form bears significant biological implications. It is known that cytisine acts as a base under physiological conditions, accepting a proton and leading to the bioactive form of the alkaloid.^12,34^ Thus, the axial or equatorial arrangement of the piperidinic nitrogen atom (N_I_), which is the protonation center (see Figure 3), plays a decisive role in the protonation process. This mechanism and the protonation energies for both conformers of cytisine were calculated in the gas and aqueous phase using a PCM model,^16^ showing that the lowest energy value is found for the protonation of the axial conformer. This fact can be easily rationalized based on the structures revealed for cytisine in the current work, as the steric hindrance for the N_I_ protonation process is lower for the axial conformer than the equatorial arrangement (see Figure 3).

**Figure 3:**
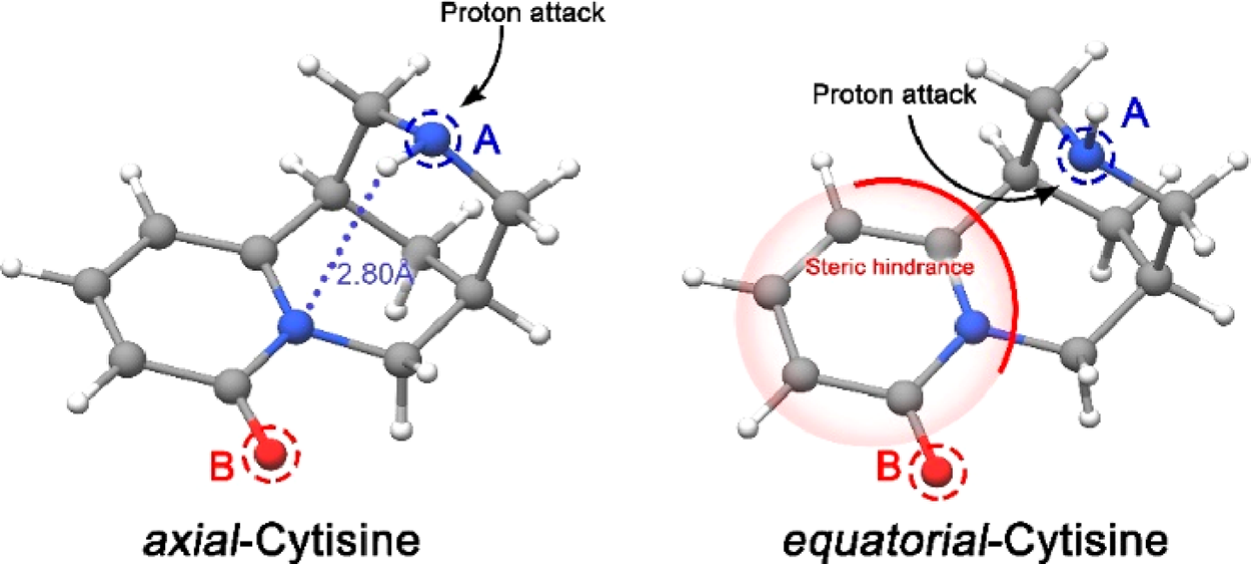
Three-dimensional structures modeled for both cytisine conformers. The N–H bond distance of 2.80 Å is highlighted for the axial conformer. We obtain an A–B distance of 4.96 Å, which agrees with the two-center model.

Finally, we can put our results in the context of the two-center model. Based on the observed structures, we can sanction the N_I_ nitrogen atom as the A center and the carbonyl oxygen atom as the B center, as these two atoms lead to an A–B distance (4.96 Å for the axial conformer; see Figure 3) that satisfies the requirement proposed for nicotinic agonists. Our results show a notorious resemblance between the shape of cytisine and nicotine. Both molecules present similar key structural motifs for the docking process with NAChRs, highlighting an almost equivalent distance between A and B centers. It further confirms the specificity of the receptor with a precise geometry of the ligand (i.e., cytisine) and the requirement of particular contact points.

In summary, we have vaporized solid cytisine by laser ablation and performed a detailed high-resolution rotational investigation. Two different axial and equatorial structures have been distinctly characterized in the supersonic jet. Surprisingly, we have observed a clear predominance of the axial form over the equatorial one. We have fully resolved the ^14^N hyperfine structure attributed to the presence of two ^14^N nuclei in the structure of cytisine using our cavity-based LA-MB-FTMW technique. It further allowed us to experimentally characterize an intramolecular NH···N hydrogen bond that overstabilizes the axial form. Interestingly, this predominant arrangement provides additional and valuable support to the two-center model that explains cytisine’s positive action over nicotinic receptors.

The marriage between laser ablation and rotational spectroscopy constitutes a unique tool to characterize the three-dimensional structure of relevant biomolecules, allowing us to scrutinize structural details not accessible to any other technique. This approach helps us to shed light on topics of biological relevance, such as explaining the role of cytisine as a nicotinic agonist.

## Supporting Information Available

Additional figure of the two detected structures for the nicotine molecule (Figure S1), detailed theoretical methodology section (section S2) with theoretical data at diferent calculation levels (Table S1) and a detailed view of the NCI and NBO calculations of cytisine molecule (Figure S2), detailed experimental section (section S3), measured frequency centers for axial (Table S2) and equatorial (Table S3) conformers of cytisine, and measured frequencies for the hyperfine components of rotational transitions belonging to the axial (Table S4) and equatorial (Table S5) conformers of cytisine (PDF)
Transparent Peer Review report available (PDF)
