# Supplementary material for: The Nicotinic Agonist Cytisine: The Role of the NH···N Interaction
Source: J Phys Chem Lett. 2022 Oct 20;13(42):9991–6. doi: 10.1021/acs.jpclett.2c02021 (PMC9619914; doi:10.1021/acs.jpclett.2c02021)
Supplement: Supplementary file 1 — jz2c02021_si_001.pdf [file jz2c02021_si_001.pdf]

Supporting information for:

The Nicotinic Agonist Cytisine: The Role Of The NH···N Interaction

Raúl Aguado[†], Santiago Mata[†], Miguel Sanz-Novo[†], Elena R. Alonso[†], Iker León[†], José L. Alonso[†,*]

[†]: Grupo de Espectroscopía Molecular (GEM), Edificio Quifima, Área de Química-Física, Laboratorios de Espectroscopía y Bioespectroscopía, Parque Científico UVa, Unidad Asociada CSIC, Universidad de Valladolid, 47011, Valladolid (Spain)

Corresponding Author

José L. Alonso. E-mail: jlalonso@qf.uva.es

S.1 Additional Figures

In Figure S1 we provide the modelled structures for the two trans conformers detected for nicotine molecule in a previous study by using high-resolution rotational spectroscopy .[1]

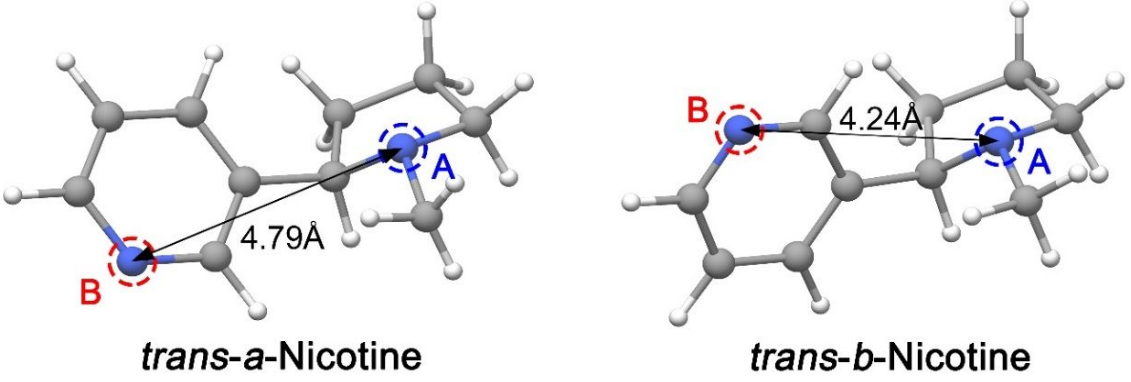

Figure S1: The two observed forms of nicotine molecule in gas phase.[1] The two proposed binding centers are highlighted. In these structures the methylated nitrogen atom acts as the proton attack site (A), and the pyridinic nitrogen atom acts as the receptor center (B). Also, we find an A-B distance that is in good accordance with the proposed model.

S.2 Theoretical Benchmark, Topological and Natural Bond Orbitals analysis

To guide the spectral search, we have computationally modelled the two plausible conformers based on the previous structural information of cytisine. In a first step , we performed a DFT calculation[2,3] using B3LYP potential[4] in combination with 6-311++G(d,p).[5] In a second step, more complex calculations were performed. Thus, we carried out B3LYP/aug-cc-pVTZ[6] combined with Grimme dispersion [7] and Becke-Johnson damping,[8] as well as MP2/6-311++G(d,p)[9] calculations. All the optimized structures were confirmed as local minima in the potential energy Surface (PES) by checking that their Hessian matrix did not have any imaginary eigenvalues. We employed the Gaussian16 package to carry out all the quantum chemical computations, [10] and the results are summarized in Table S1.

To characterize conclusively the NH···N interaction which is most likely present in the predominant axial-conformer, we used the wavefunction of the refined structures optimized at the B3LYP/aug-cc-pVTZ level to carry out non-covalent interactions (NCI) computations.[11] By using NCIPLOT 4 software [12] a representation of the different intramolecular non-covalent interactions of both conformers has been obtained (see Figure S1[a]). Moreover, we have performed an analysis of Natural Bonding Orbitals (NBOs)[13] of the system (Figure S1[b]). The results of the NCI analysis revealed a stabilizing interaction involving the piperidinic N_I nucleus in both conformers which is strong for the axial conformer (as there are no Type II isosurfaces between both N nuclei). Additionally, more detailed information regarding these interactions can be extracted from the examination of the Natural Bonding Orbitals (NBOs) calculations. This methodology shows that for the axial conformer there is an interaction between the piperidinic NH bonding orbital and the lone pair of N_{III} nucleus, further corroborating the experimentally detected NH···N interaction. In the other hand, for equatorial conformer, the NBOs analysis points to an electrostatic interaction between the lone pair of N_I and N_{III} nuclei, which has been demonstrated experimentally to be much less stabilizing than the NH···N hydrogen bond.

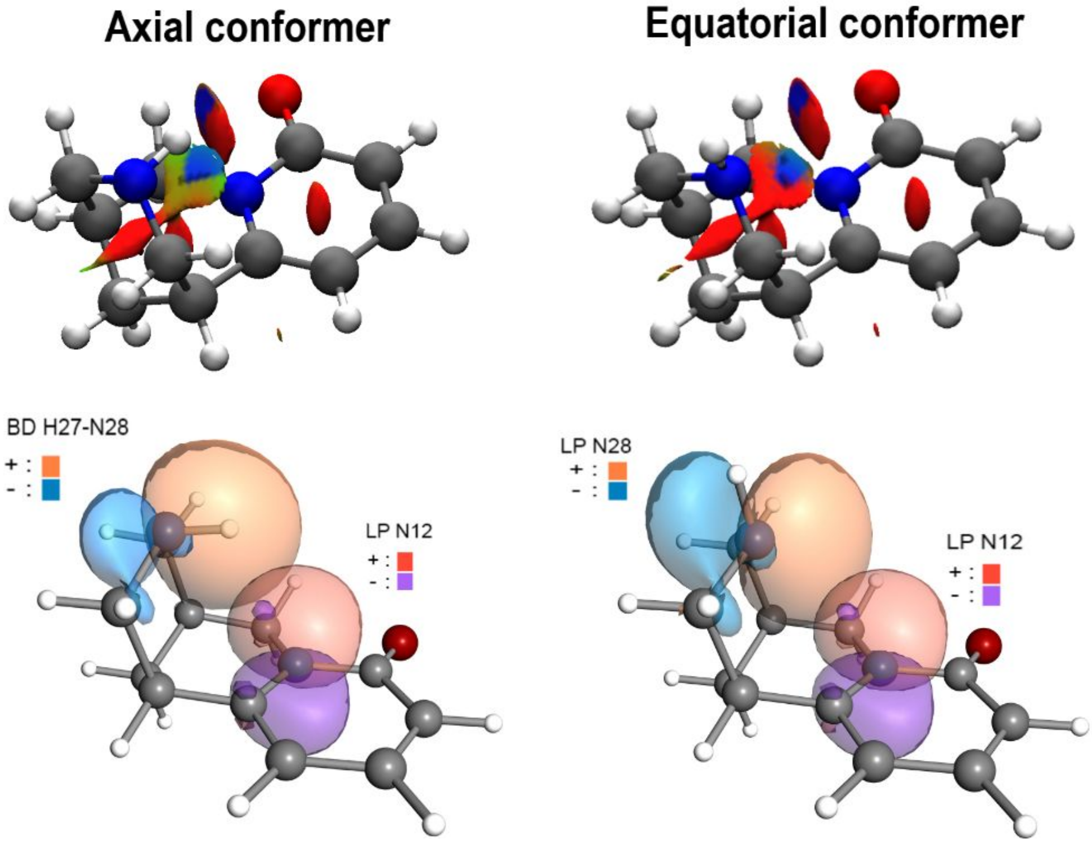

Figure S2. (a): Visual molecular dynamics (VMD) graphics of cytisine NCI analysis.[14] Different NCI type I, II and III isosurfaces were found. NCI type I isosurfaces (blue) correspond to strong stabilizing interactions, NCI type II isosurfaces (red) account for strong destabilizing interactions, and NCI type III (green) represent delocalized moderately strong van der Waals interactions.[15] (b): NBOs representation for both conformers of cytisine.

Table S1. Theoretical prediction of spectroscopic parameters for both plausible conformers of cytisine

	Axial			Equatorial		
	**B3LYP 6-311++G(d,p)**	**B3LYP aug-cc-pVTZ**	**MP2 6-311++G(d,p)**	**B3LYP 6-311++G(d,p)**	**B3LYP aug-cc-pVTZ**	**MP2 6-311++G(d,p)**
A[a]	1235	1241	1233	1247	1253	1247
B	645	647	652	643	645	651
C	517	519	521	514	515	519
μ_a [b]	2.8	2.8	2.5	4.6	4.5	4.4
μ_b	2.9	2.8	2.7	3.3	3.2	3.2
μ_c	1.6	1.5	1.7	0.6	0.6	0.6
χ_{aa} (N_{III}) [c]	0.973	0.9170	0.719	1.057	0.989	0.788
χ_{bb} (N_{III})	1.700	1.5594	1.453	1.704	1.563	1.458
χ_{cc} (N_{III})	-2.673	-2.4764	-2.172	-2.762	-2.552	-2.246
χ_{aa} (N_I)	-1.209	-1.2872	-0.848	-5.135	-4.937	-4.669
χ_{bb} (N_I)	2.852	2.7780	2.653	2.678	2.618	2.432
χ_{cc} (N_I)	-1.643	-1.4908	-1.805	2.457	2.319	2.237
ΔE[c]	0	0	0	210.9	148.8	0.4
ΔE_{ZPE}	0	0	0	211.4	151.4	6.8
ΔG	0	0	0	218.6	161.3	29.6

[a]: *A, B, C* are the rotational constants (in MHz); [b]: $|\mu_a|$, $|\mu_b|$ and $|\mu_c|$ are the absolute values of the dipole moment (in Debyes); [c]: χ_{aa}, χ_{bb}, χ_{cc} are the ^{14}N nuclear quadrupole coupling constants (in MHz); [d]: ΔE are energies relative to the global minimum; [e]: ΔE_{ZPE} are energies relative to the global minimum taking into account the zero-point energy (ZPE); [f]: are the Gibbs energies relative to the global minimum calculated at 298 K; All energies are expressed in cm^{-1}

S.3 Experimental methodology

LA-CP-FTMW spectroscopy

The broad rotational spectrum of cytisine has been investigated using two different LA-CP-FTMW spectrometers operating in the 3–6 GHz and the 6-14GHz frequency region.[16,17] A commercial sample of cytisine (99%, m.p. 156°C) was used without any further purification to form solid rods by pressing the compound's fine powder mixed with a small amount of a commercial copolymeric binder. One of this rods was placed in the ablation nozzle of our 2–6 GHz spectrometer and a picosecond Nd:YAG laser (12 mJ per pulse, 35 ps pulse width, 355nm) was used to transfer the cytisine molecules to the gas phase. The resulting products of the laser ablation process were seeded in Ne (backing pressure of 10 bar), supersonically expanded and finally probed by chirped-pulse Fourier-transform microwave spectroscopy. We employed chirped-pulses of 4 ms, generated by a 24 GS·s^{-1} arbitrary waveform generator, that were amplified using a 200 W solid state amplifier. Two microwave horns were used to broadcast the excitation pulse and receive the broadband molecular emission. At a repetition rate of 2 Hz, a

total of 90 000 free induction decays were averaged and digitized using a 25 GS·s-1 digital oscilloscope. Finally, the time-domain spectrum was Fourier-transformed after applying a Kaiser-Bessel window to obtain the broadband spectrum in the frequency-domain.

To obtain the spectrum in the 6-14 GHz region, a fresh sample rod was placed in the ablation nozzle of the second spectrometer. Again, a picosecond Nd:YAG laser (12 mJ per pulse, 35 ps pulse width, 355nm) was used to transfer the cytisine molecules to the gas phase, which were seeded in Ne at a backing pressure of 10 bar and supersonically expanded into the vacuum chamber. A 24 GS·s^{-1} arbitrary waveform generator was used to generate chirped-pulses of 4 ms that were further amplified by a 300W TWT amplifier. Inside the chamber, two parabolic reflector in a paraxial configuration were used to broadcast the excitation pulses and to receive the molecular FIDs. Up to 90 000 free induction decays were averaged and digitized using a 25 GS·s-1 digital oscilloscope and, finally, Fourier-transformed to obtain the spectrum in the frequency-domain.

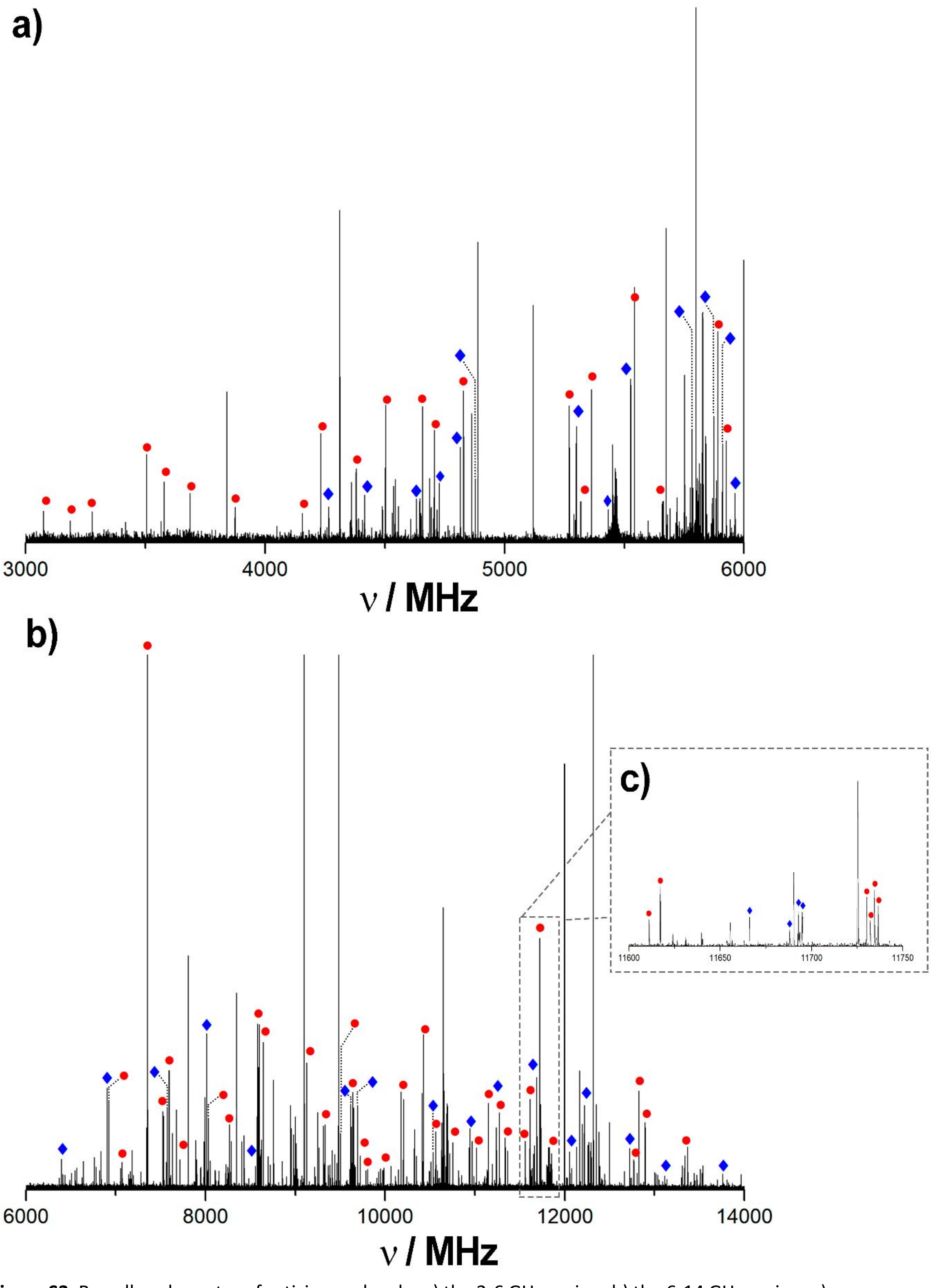

Figure S3. Broadband spectra of cytisine molecule. a) the 3-6 GHz region; b) the 6-14 GHz region; c) zoom-in to the 11.50-11.75 GHz.

LA-MB-FTMW spectroscopy

The complex hyperfine structure originated from two ^{14}N nuclei cannot be easily resolved using a broadband technique. Thus, in a second step, we exploited the high resolution of our molecular-beam Fourier-transform spectrometer[18] in combination with a laser ablation source (Nd:YAG picosecond laser, 14 mJ per pulse, 35 ps pulse width, 355nm) as vaporization tool. The ablated molecules were seeded in a carrier gas (Ne, backing pressure of 10 bar) and supersonically expanded into a Fabry-Pérot resonator. A short microwave radiation pulse was applied to macroscopically polarize all the vaporized molecules. The free induction decay was then registered and Fourier-transformed into the frequency domain. The parallel orientation of the molecular beam with respect to the microwave radiation implies that all the transitions will appear as Doppler doublets. The resonance frequency can be, therefore, determined as the arithmetic mean of the two Doppler components.

S.4 Measured transitions in the rotational spectrum

In Tables S2-S5 we provide the measured transitions for the axial and equatorial conformers of cytisine obtained through the analysis of LA-CP-FTMW spectrum (Table S2 and S3) or through the analysis of the LA-MB-FTMW spectrum (Table S4 and S5), respectively.

Table S2. Measured frequencies for the rotational transitions of the axial conformer of cytisine. The experimental frequencies are obtained measuring the center of frequencies of the broaden lines in LA-CP-FTMW spectrum.

J’	K'_{-1}	K'_{+1}	J’’	K''_{-1}	K''_{+1}	ν_{obs} / MHz	$\nu_{obs}-\nu_{calc}$ / MHz
3	3	1	3	2	2	3278.02	-0.03
3	1	3	2	1	2	3299.79	0.10
7	1	6	7	0	7	3343.99	0.00
3	2	2	2	2	1	3505.21	0.01
3	1	2	2	1	1	3686.59	0.05
7	2	6	7	1	7	3874.36	0.05
4	1	4	3	1	3	4381.08	0.04
4	0	4	3	0	3	4503.20	0.09
4	4	1	4	3	1	4531.59	0.04
4	2	3	3	2	2	4658.59	-0.06
4	3	2	3	3	1	4707.10	0.04
5	1	4	4	2	3	4708.33	-0.01
4	3	1	3	3	0	4717.69	0.06
4	1	3	3	1	2	4888.68	0.03
3	2	2	2	1	1	5271.10	0.10

5	0	5	4	1	4	5318.85	0.01
5	1	5	4	1	4	5450.87	-0.02
5	0	5	4	0	4	5543.42	0.09
5	5	0	5	4	1	5839.80	-0.06
5	5	1	5	4	2	5841.02	-0.06
5	4	2	4	4	1	5884.47	0.06
5	4	1	4	4	0	5885.58	0.07
5	3	3	4	3	2	5891.24	0.10
5	3	2	4	3	1	5926.99	0.02
3	3	1	2	2	0	6764.20	0.08
3	3	1	2	2	1	6783.27	0.02
3	3	0	2	2	1	6784.99	-0.04
6	2	5	5	2	4	6925.66	0.03
6	3	4	5	3	3	7073.34	0.00
6	4	2	5	4	1	7078.82	0.05
6	2	4	5	2	3	7360.77	0.01
7	0	7	6	1	6	7524.90	0.05
7	0	7	6	0	6	7597.10	0.04
5	2	3	4	1	3	7715.00	0.05
7	2	6	6	2	5	8035.32	-0.02
7	3	5	6	3	4	8249.06	0.02
7	4	3	6	4	2	8283.89	0.03
7	1	6	6	1	5	8286.58	0.02
8	0	8	7	1	7	8589.93	0.04
7	2	5	6	2	4	8601.38	0.00
8	1	8	7	1	7	8608.59	-0.03
8	0	8	7	0	7	8627.44	0.08
8	1	8	7	0	7	8646.14	0.04
8	2	7	7	2	6	9128.56	-0.01
8	1	7	7	1	6	9332.93	0.10
8	3	6	7	3	5	9413.59	-0.04

8	4	5	7	4	4	9465.32	-0.02
8	4	4	7	4	3	9506.60	0.05
9	0	9	8	1	8	9642.40	0.03
9	1	9	8	1	8	9651.54	0.04
9	0	9	8	0	8	9661.07	-0.04
9	1	9	8	0	8	9670.18	-0.05
8	2	6	7	2	5	9807.13	-0.02
9	1	8	8	2	7	9989.66	0.06
9	2	8	8	2	7	10206.56	-0.01
5	4	2	4	3	1	10415.96	0.00
5	4	1	4	3	1	10417.21	0.01
5	4	2	4	3	2	10428.36	0.04
5	4	1	4	3	2	10429.58	0.02
9	3	7	8	3	6	10563.20	-0.05
9	4	6	8	4	5	10662.27	0.04
10	0	10	9	1	9	10688.06	0.03
10	1	10	9	1	9	10692.41	0.03
10	0	10	9	0	9	10697.18	0.03
10	1	10	9	0	9	10701.55	0.05
9	4	5	8	4	4	10754.17	0.01
9	2	7	8	2	6	10968.91	0.01
9	3	6	8	3	5	11021.06	0.04
10	1	9	9	2	8	11149.34	0.01
10	2	9	9	2	8	11271.72	-0.04
10	1	9	9	1	8	11366.32	0.01
6	4	3	5	3	2	11562.94	0.02
6	4	3	5	3	3	11611.11	0.00
6	4	2	5	3	3	11617.19	0.00
11	0	11	10	1	10	11730.19	0.02
11	1	11	10	1	10	11732.24	0.04
11	0	11	10	0	10	11734.54	0.03

11	1	11	10	0	10	11736.56	0.02
10	5	6	9	5	5	11844.78	-0.01
10	4	7	9	4	6	11854.52	0.01
10	4	6	9	4	5	12032.76	-0.02
11	1	10	10	2	9	12260.91	-0.01
8	3	5	7	2	5	12261.20	-0.01
10	3	7	9	3	6	12299.33	0.01
11	2	10	10	2	9	12327.04	-0.06
11	1	10	10	1	9	12383.34	-0.01
11	2	10	10	1	9	12449.49	-0.03
12	0	12	11	1	11	12770.56	0.03
12	1	12	11	1	11	12771.50	0.03
12	0	12	11	0	11	12772.59	0.03
12	1	12	11	0	11	12773.54	0.03
7	4	3	6	3	4	12827.74	0.02
6	5	2	5	4	1	12898.49	-0.01
6	5	1	5	4	2	12899.81	-0.03
11	4	8	10	4	7	13037.35	-0.02
11	4	7	10	4	6	13340.88	0.01
12	2	11	11	2	10	13375.53	-0.02
12	2	11	11	1	10	13441.75	0.03
9	3	6	8	2	6	13475.08	0.01
11	3	8	10	3	7	13541.04	-0.05

Table S3. Measured frequencies for the rotational transitions of the equatorial conformer of cytisine. The experimental frequencies are obtained measuring the center of frequencies of the broaden lines in LA-CP-FTMW spectrum.

J'	K'_{-1}	K'_{+1}	J''	K''_{-1}	K''_{+1}	ν_{obs} / MHz	$\nu_{obs} - \nu_{calc}$ / MHz
2	2	1	1	1	0	4266.02	-0.10
2	2	0	1	1	1	4415.86	0.07
4	2	3	3	2	2	4646.20	0.02
4	3	2	3	3	1	4694.40	-0.07

4	1	4	3	0	3	4723.04	-0.07
4	2	2	3	2	1	4816.28	-0.02
4	1	3	3	1	2	4878.38	-0.15
5	0	5	4	1	4	5295.12	-0.08
3	2	2	2	1	1	5300.67	-0.10
5	1	5	4	1	4	5432.56	-0.09
5	0	5	4	0	4	5527.52	0.05
5	1	5	4	0	4	5665.08	0.17
5	2	4	4	2	3	5784.11	0.01
3	2	1	2	1	2	5785.43	0.09
5	4	2	4	4	1	5868.68	0.07
5	4	1	4	4	0	5869.75	0.08
5	3	3	4	3	2	5875.52	0.05
5	3	2	4	3	1	5910.76	-0.01
5	5	0	5	4	1	5962.36	0.01
6	0	6	5	1	5	6412.98	0.01
6	1	6	5	1	5	6626.17	0.04
6	2	5	5	2	4	6907.12	0.06
6	2	4	5	2	3	7343.42	0.06
7	1	7	6	1	6	7536.59	0.01
7	0	7	6	0	6	7572.77	0.05
7	1	7	6	0	6	7612.27	0.03
7	2	6	6	2	5	8013.81	0.01
8	0	8	7	1	7	8559.02	-0.05
8	1	8	7	1	7	8578.94	-0.01
8	0	8	7	0	7	8598.59	0.01
8	1	8	7	0	7	8618.50	0.03
8	2	7	7	2	6	9104.08	0.07
9	0	9	8	1	8	9608.06	-0.01
9	1	9	8	1	8	9617.80	0.00
9	0	9	8	0	8	9627.93	-0.03

9	3	7	8	3	6	10536.50	-0.06
10	0	10	9	1	9	10649.96	0.01
10	1	10	9	1	9	10654.63	0.02
10	0	10	9	0	9	10659.69	0.01
10	1	10	9	0	9	10664.36	0.01
9	4	5	8	4	4	10723.63	0.01
9	2	7	8	2	6	10948.28	0.00
10	2	9	9	2	8	11240.74	0.00
10	1	9	9	1	8	11339.62	0.06
10	3	8	9	3	7	11666.16	-0.02
11	0	11	10	1	10	11688.10	0.00
11	1	11	10	1	10	11690.30	0.00
11	0	11	10	0	10	11692.76	0.00
10	2	8	9	2	7	12058.34	0.01
11	1	10	10	2	9	12221.81	-0.05
10	3	7	9	3	6	12270.24	0.00
11	2	10	10	2	9	12292.55	-0.03
11	1	10	10	1	9	12351.85	0.02
12	0	12	11	1	11	12724.41	0.02
12	1	12	11	1	11	12725.41	-0.01
12	0	12	11	0	11	12726.59	0.00
12	1	12	11	0	11	12727.61	-0.01
11	3	9	10	3	8	12777.06	-0.03
11	2	9	10	2	8	13116.76	-0.01
12	2	11	11	2	10	13337.27	-0.04
12	1	11	11	1	10	13370.77	-0.03
13	0	13	12	1	12	13759.76	-0.02
13	1	13	12	1	12	13760.22	-0.03
13	0	13	12	0	12	13760.81	0.01

Table S4. Measured frequencies for the hyperfine components of rotational transitions belonging to the axial conformer of cytisine obtained using LA-MB-FTMW spectroscopy.

J’	K'_{-1}	K'_{+1}	F’	I’	J’’	K''_{-1}	K''_{+1}	F’’	I’’	ν_{obs} / MHz	$\nu_{obs} - \nu_{calc}$ / MHz
4	0	4	4	4	3	1	3	3	3	4156.284	-0.003
4	0	4	5	5	3	1	3	4	4	4156.416	0.001
4	0	4	4	5	3	1	3	3	4	4156.479	0.000
4	0	4	4	3	3	1	3	3	2	4156.538	0.000
4	0	4	5	6	3	1	3	4	5	4156.650	0.000
4	0	4	5	4	3	1	3	4	3	4156.738	0.000
4	0	4	3	4	3	1	3	2	3	4156.758	-0.001
5	0	5	5	5	4	1	4	4	4	5318.740	0.000
5	0	5	5	6	4	1	4	4	5	5318.830	0.000
5	0	5	6	6	4	1	4	5	5	5318.791	0.003
5	0	5	6	7	4	1	4	5	6	5318.896	0.001
3	2	1	4	4	2	1	1	2	3	5364.030	0.001
3	2	1	3	4	2	1	1	3	3	5364.415	0.001
3	2	1	3	3	2	1	1	2	2	5364.474	0.002
5	0	5	5	5	4	0	4	4	4	5543.607	-0.003
5	0	5	4	3	4	0	4	3	2	5543.221	-0.001

Table S5. Measured frequencies for the resolved hyperfine rotational transitions of the equatorial conformer of cytisine obtained using LA-MB-FTMW spectroscopy.

J’	K'_{-1}	K'_{+1}	F’	I’	J’’	K''_{-1}	K''_{+1}	F’’	I’’	ν_{obs} / MHz	
3	1	3	3	3	2	1	2	3	2	3288.735	-0.001
3	1	3	4	3	2	1	2	2	2	3288.686	-0.003
3	1	3	3	2	2	1	2	2	1	3289.014	0.000
4	0	4	5	4	3	1	3	4	3	4134.080	-0.001
4	0	4	5	6	3	1	3	4	5	4134.207	0.004
4	0	4	5	6	3	1	3	4	5	4490.818	0.001
4	0	4	4	3	3	1	3	3	2	4490.906	-0.004

4	0	4	4	5	3	1	3	3	4	4491.033	0.001
5	0	5	6	7	4	1	4	5	6	5295.198	-0.006
5	0	5	6	5	4	1	4	5	4	5527.413	0.001
5	0	5	6	7	4	1	4	5	6	5527.487	0.000
5	0	5	5	4	4	1	4	4	3	5527.609	-0.002
5	1	5	5	4	4	0	4	4	3	5665.204	0.004
4	1	4	5	6	3	1	3	4	5	4366.490	0.003
4	1	4	3	4	3	1	3	2	3	4366.543	-0.003
4	1	4	4	5	3	1	3	3	4	4366.602	0.004
4	1	4	5	5	3	1	3	4	4	4366.314	0.004
5	1	5	6	6	4	1	4	5	5	5432.595	-0.001
5	1	5	6	7	4	1	4	5	6	5432.683	-0.003
5	1	5	5	6	4	1	4	4	5	5432.781	0.002

S.5 References

[1] J. U. Grabow, S. Mata, J. L. Alonso, I. Peña, S. Blanco, J. C. López, C. Cabezas. A Broadband Fourier-Transform Microwave Spectrometer With Laser Ablation Source: The Rotational Spectrum Of Nicotinic Acid. *Phys. Chem. Chem. Phys.* **2011**, *13*, 21063–21069.

[2] A. D. Becke. Density-Functional Exchange-Energy Approximation With Correct Asymptotic Behavior. *Phys. Rev. A* **1988**, *38*, 3098–3100.

[3] A. D. Becke. A New Mixing Of Hartree-Fock And Local Density-Functional Theories. *J. Chem. Phys.* **1993**, *98*, 1372–1377.

[4] C. Lee, W. Yang, R. G. Parr. Self-Consistent Molecular Orbital Methods 25. Supplementary Functions For Gaussian Basis Sets. *Phys. Rev. B* **1988**, *37*, 785–789.

[5] M. J. Frisch, J. A. Pople, J. S. Binkley. Self-Consistent Molecular Orbital Methods 25. Supplementary Functions For Gaussian Basis Sets. *J. Chem. Phys.* **1984**, *80*, 3265–3269.

[6] T. H. Dunning. Gaussian Basis Sets For Use In Correlated Molecular Calculations. I. The Atoms Boron Through Neon And Hydrogen. *J. Chem. Phys.* **1989**, *90*, 1007–1023.

[7] T. Schwabe, S. Grimme. Double-Hybrid Density Functionals With Long-Range Dispersion Corrections: Higher Accuracy And Extended Applicability. *Phys. Chem. Chem. Phys.* **2007**, *9*, 3397–3406.

[8] S. Grimme, S. Ehrlich, L. Goerigk. Effect Of The Damping Function In Dispersion Corrected Density Functional Theory. *J. Comput. Chem.* **2011**, *32*, 1456–1465.

[9] C. Møller, M. S. Plesset. Note On An Approximation Treatment For Many-Electron Systems. *Phys. Rev.* **1934**, *46*, 618–622.

[10] M. J. Frisch, G. W. Trucks, H. B. Schlegel, G. E. Scuseria, M. A. Robb, J. R. Cheeseman, G. Scalmani, V. Barone, G. A. Petersson, H. Nakatsuji, X. Li, M. Caricato, A. V. Marenich, J. Bloino, B. G. Janesko, R. Gomperts, B. Mennucci, H. P. Hratchian, J. V. Ortiz, A. F. Izmaylov, J. L. Sonnenberg, D. Williams-Young, F. Ding, F. Lipparini, F. Egidi, J. Goings, B. Peng, A. Petrone, T. Henderson, D. Ranasinghe, V. G. Zakrzewski, J. Gao, N. Rega, G. Zheng, W. Liang, M. Hada, M. Ehara, K. Toyota, R. Fukuda, J. Hasegawa, M. Ishida, T. Nakajima, Y. Honda, O. Kitao, H. Nakai, T. Vreven, K. Throssell, J. A. Montgomery Jr., J. E. Peralta, F. Ogliaro, M. J. Bearpark, J. J. Heyd, E. N. Brothers, K. N. Kudin, V. N. Staroverov, T. A. Keith, R. Kobayashi, J. Normand, K. Raghavachari, A. P. Rendell, J. C. Burant, S. S. Iyengar, J. Tomasi, M. Cossi, J. M. Millam, M. Klene, C. Adamo, R. Cammi, J. W. Ochterski, R. L. Martin, K. Morokuma, O. Farkas, J. B. Foresman, D. J. Fox. Gaussian 16. **2016**.

[11] C. Narth, Z. Maroun, R. A. Boto, R. Chaudret, M.-L. Bonnet, J.-P. Piquemal, J. Contreras-García. A Complete NCI Perspective: From New Bonds to Reactivity in *Appl. Topol. Methods Mol. Chem.* (Eds.: R. Chauvin, C. Lepetit, B. Silvi, E. Alikhani), Springer International Publishing, Cham, **2016**, pp. 491–527.

[12] J. Contreras-García, E. R. Johnson, S. Keinan, R. Chaudret, J.-P. Piquemal, D. N. Beratan, W. Yang. NCIPLOT: A Program for Plotting Noncovalent Interaction Regions. *J. Chem. Theory Comput.* **2011**, *7*, 625–632.

[13] F. Weinhold, C. R. Landis. Natural Bond Orbitals and Extensions of Localized Bonding Concepts. *Chem. Educ. Res. Pr.* **2001**, *2*, 91–104.

[14] W. Humphrey, A. Dalke, K. Schulten. VMD: Visual Molecular Dynamics. *J. Mol. Graph.* **1996**, *14*, 33–38.

[15] N. Gillet, R. Chaudret, J. Contreras-García, W. Yang, B. Silvi, J.-P. Piquemal. Coupling Quantum Interpretative Techniques: Another Look at Chemical Mechanisms in Organic Reactions. *J. Chem. Theory Comput* **2012**, *8*, DOI 10.1021/ct300234g.

[16] E. R. Alonso, Biomolecules and Interstellar Molecules: Structure, Interactions and Spectroscopic Characterization. Universidad de Valladolid, **2018**.

[17] I. Peña, S. Mata, A. Martín, C. Cabezas, A. M. Daly, J. L. Alonso. Conformations Of D-Xylose: The Pivotal Role Of The Intramolecular Hydrogen-Bonding. *Phys. Chem. Chem. Phys.* **2013**, *15*, 18243–18248.

[18] M. E. Sanz, C. Cabezas, S. Mata, J. L. Alonso. Rotational Spectrum Of Tryptophan. *J. Chem. Phys.* **2014**, *140*, DOI 10.1063/1.4876001.
