# Supplementary material for: The Nicotinic Agonist Cytisine: The Role of the NH···N Interaction
Source: J Phys Chem Lett. 2022 Oct 20;13(42):9991–6. doi: 10.1021/acs.jpclett.2c02021 (PMC9619914; doi:10.1021/acs.jpclett.2c02021)
Supplement: Supplementary file 2 — jz2c02021_si_002.pdf [file jz2c02021_si_002.pdf]

jz-2022-020214.R1

Name: Peer Review Information for "The Nicotinic Agonist Cytisine: The Role Of The NH···N Interaction"

First Round of Reviewer Comments

Reviewer: 1

Comments to the Author

This paper represents a microwave rotational study of the molecule cytosine to determine the gas phase structure of the molecule as it pertains to both the organic and biochemical fields as a possible cessation approach to nicotine. The conclusions of this work focus on the determination of the axial form of cystine to be the most stable structure. This, as the authors claim, would be very interesting and unusual and important to the structural community. However, there are a lot of questions surrounding this conclusion and many concerns need to be addressed before any recommendation for publication can be considered.

1. The authors detail the axial form as "predominate" in their spectra. This, simply, is not supported by the evidence presented in the paper. This is because the authors only present 3 GHz of spectra consisting of <50 total measured lines with each technique with a-, b-, and c-types being observable for the axial and not really as strong for the equatorial. Therefore, the only way I see this claim being made is heavily relying on the theoretical data provided, which does support that claim.

2. Why only 3 GHz of spectra? The authors have access to much more spectral capacity to measure lines than this. This may help support their claim from my first issue.

3. The comparison of rotational constants and quadrupole coupling constants, in my opinion, does not really help with the determination of Rotamer I and II. However, the authors do not present anywhere a determination of the centrifugal distortion constants. This could also be used to compare to theory to help with the structure determination, but was not presented.

4. It is unclear to me that the method for creating the gas phase cytosine, laser ablation, would give a definitive answer as to the lower energy structure. Laser vaporization sources are known for providing a lot of energy to the system. The mechanistic pathway to the bottom of the well is then reliant on the expansion gas utilized and how it goes about cooling the resultant species. The authors already claim there are products due to photofragmentation, implying a lot of introduced energy, could they provide information on how the relaxation process takes place to ensure the relaxation to the global minimum?

Reviewer: 2

Comments to the Author

The authors investigated the cytisine by means of rotational spectroscopy, identifying the axial and equatorial conformer. Contrary to what one might think, the axial conforme is more stable and they managed to motivate the experimental evidence. The experimental work is certainly well developed, the results are widely supported by the data that are original and coherent. The computational part is also well described and amply supports the results obtained.

My concerns arise from the context developed within the article. The authors speak of Nicotinic Agonist Cytisine bringing as references some works including the famous:

Sheridan, R. P .; Nilakantan, R .; Dixon, J. S .; Venkataraghavan, R. The Ensemble

Approach to Distance Geometry: Application to the Nicotinic Pharmacophore. J. Med.

Chem. 1986, 29 (6), 899–906. https://doi.org/10.1021/jm00156a005.

However in that article they speak of a three-center pharmacophore while the authors continue to highlight only a two-center model (A - B). In this context the three-dimensionality is lost and it is not clear whether the molecule actually reflects the selected criteria.

Furthermore, the discussion also focuses on the subsequent protonation of the molecule. But what would be the difference between the axial and the equatorial form once protonated? The two conformers would be the same without any distinction.

Then there are some typos that authors should take into consideration:

1) Page 5: I should be italics

2) Figure 2: I and F shoul be italics

3) Table 1: They wrote B3LYP. Is it right? Or they use an empirical dispersion?

4) Page 10: NI-H, write I in pedix

Reviewer: 3

Comments to the Author

The paper reports microwave spectroscopic results of cytosine, an alkaloid with biological activity. Although the molecule is important for its biological activity, the size of the molecule is fairly large for detailed spectroscopic investigation. The authors group has been successfully obtained such molecules in the gas phase and characterized them by rotational spectroscopy. They were able to detect two conformers for this molecule determining relative abundances between them, based on quantum chemical calculations. This result is a key to discuss its biological activity. Thus, the paper contains unique new information not available by other methods, and worth publishing.

Author's Response to Peer Review Comments:

August 18th, 2022

Dear Prof. Editor,

A revised copy of our manuscript (ID: jz-2022-020214) entitled: "The Nicotinic Agonist Cytisine: the Role of the NH···N Interaction" has been uploaded.

We appreciate this opportunity to have our work considered in The Journal of Physical Chemistry Letters, and we are grateful to the reviewers for their comments. Following their suggestions, we have made some changes in the paper that we detail below. Our comments to the referees are remarked in italics, and the corresponding changes made in the manuscript are highlighted in bold. Thanks to the reviewers' comments, the new version has improved quality.

Moreover, we have double-checked the non-scientific changes addressed by in the previous letter (title and references format, etc…).

Best regards,

Prof. José Luis Alonso

We thank reviewer 1 for his/her comments. Following his /her suggestions, we have made the corresponding changes in the manuscript that are here indicated between quotations marks and also highlighted in bold in the revised manuscript.

Reviewer 1:

This paper represents a microwave rotational study of the molecule cytosine to determine the gas phase structure of the molecule as it pertains to both the organic and biochemical fields as a possible cessation approach to nicotine. The conclusions of this work focus on the determination of the axial form of cystine to be the most stable structure. This, as the authors claim, would be very interesting and unusual and important to the structural community. However, there are a lot of questions surrounding this conclusion and many concerns need to be addressed before any recommendation for publication can be considered.

1. The authors detail the axial form as "predominate" in their spectra. This, simply, is not supported by the evidence presented in the paper. This is because the authors only present 3 GHz of spectra consisting of <50 total measured lines with each technique with a-, b-, and c-types being observable for the axial and not really as strong for the equatorial. Therefore, the only way I see this claim being made is heavily relying on the theoretical data provided, which does support that claim.

(Reply) "The referee is right that a priori, a 3 GHz spectral range could seem a bit limited. However, the study of large fairy molecules employing rotational spectroscopy is usually carried out from 2.0-8.0 GHz (in this case, up to 6.250 GHz due to the frequency limitations of the solid-state amplifier), where the rotational levels present the maximum population in the supersonic expansion at the characteristic low temperatures of these experiments (less than 10K). Additionally, for systems exhibiting a relatively large size, the rotational constants' values are also low; therefore, a relatively "short" spectral range is usually enough to measure a sufficient number of rotational transitions. Thus, the spectral window tackled in the current work does provide enough experimental evidence for the scientific findings of our investigation.

Nevertheless, following the reviewer's suggestions, we have extended the frequency range to 14 GHz using a TWT amplifier. It has allowed us to measure a considerably large number of rotational transitions. With such results, the values of the rotational constants are more accurate, and, particularly, the information about the relative populations of both conformers (axial form being predominant) is very confident, as explained below.

Furthermore, to conclusively assert the predominance of the axial form, we derived the relative abundance between axial and equatorial conformers by following a similar strategy as that reported in J. Mol. Spectrosc. 2012, 280, 77–84. We used a controlled polarization power to try to ensure a linear fast passage polarization regime, for which the intensity of a transition is proportional to the square of the dipole moment. In this context, we selected a- and b-type R-branch lines very close in frequency (same J values), which should have been polarized with the same fast passage polarization regime, to perform relative intensity measurements. We then computed the intensities of the selected set of transitions and corrected them considering the corresponding computed dipole moment components. Thus, we have added two new references [Schmitz, D., Shubert, V. A., Betz, T. Schnell, M., J. Mol. Spectrosc. 2012, 280, 77–84 and references therein; G. T. Fraser, R. D. Suenram, C. L. Lugez, J. Phys. Chem. A 2001, 105, 9859-9864 references therein] to describe relative intensity measurements. Other groups commonly

use similar strategies (Chem. Commun. 2015, 51, 10115-10118; Phys. Chem. Chem. Phys. 2019, 21, 15408; J. Phys. Chem. Lett. 2021, 12, 1081-1086; J. Chem. Phys. 2015, 142, 104309)."

Additionally, considering the reviewer's comment, we have added a new Figure S2 in the Supporting Information showing the complete spectrum between 2 to 6 GHz and 6-14GHz so that any reader can have a complete view of the overall predominance of the axial form."

Accordingly, we have modified Table 1 and Tables S2 and S3, including the new data.

2. Why only 3 GHz of spectra? The authors have access to much more spectral capacity to measure lines than this. This may help support their claim from my first issue.

(Reply) "Following the reviewer's previous point, we have extended the broadband range. The spectrum in the 6-14 GHz region is shown in Figure S3 and is also included below. The extension of the broadband range has allowed us to measure up to 100 transitions for axial rotamer and 56 transitions for equatorial conformer. It has improved the rotational constants' values. The new data shown below have been included in Table 1.

	**Theoretical** B3LYP-GD3/aug-cc-pVTZ		**LA-CP-FTMW** spectroscopy	
Parameter	**Axial**	**Equatorial**	**Rotamer I**	**Rotamer II**
A[a]	1241.4	1253.1	1237.5720(33)[g]	1249.5815(98)
B	647.3	645.2	648.9721(15)	647.9141(33)
C	518.8	515.8	519.42718(78)	517.3335(10)
$\|\mu_a\|$[b]	2.8	4.6	Yes	Yes
$\|\mu_b\|$	2.9	3.3	Yes	Yes
$\|\mu_c\|$	1.6	0.6	Yes	No
χ_{aa} (N_{III}) [c]	0.9170	0.989	Non-resolved using this technique	
χ_{bb} (N_{III})	1.5594	1.563		
χ_{cc} (N_{III})	-2.4764	-2.552		
χ_{aa} (N_I)	-1.2872	-4.937		
χ_{bb} (N_I)	2.7780	2.618		
χ_{cc} (N_I)	-1.4908	2.319		

σ_{rms} [d]	-	-	41.5	39.8
N[e]	-	-	100	56

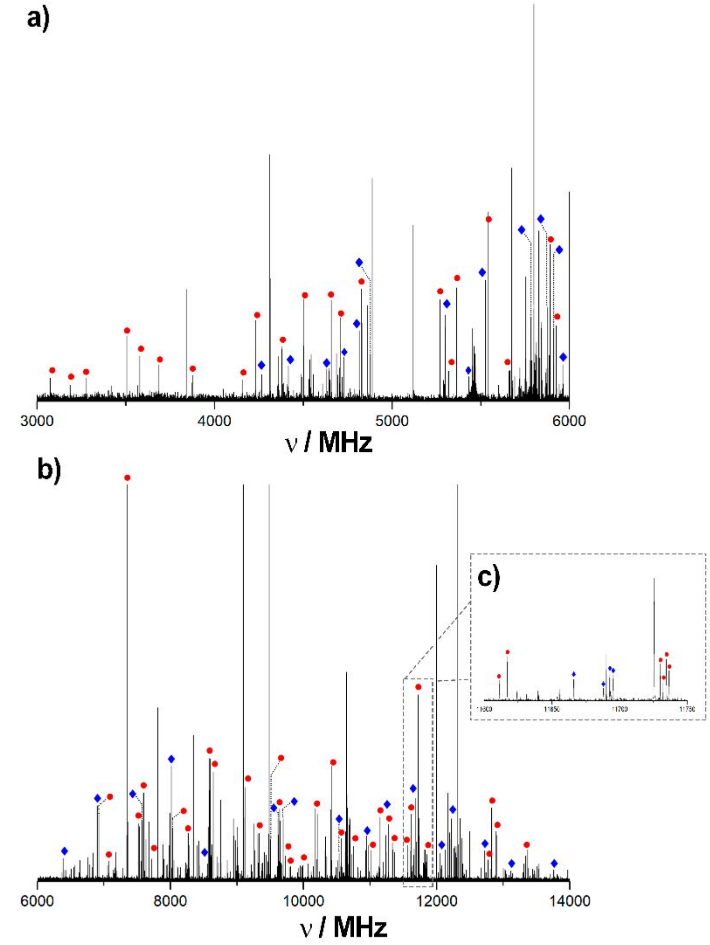

3. The comparison of rotational constants and quadrupole coupling constants, in my opinion, does not really help with the determination of Rotamer I and II. However, the authors do not present anywhere a determination of the centrifugal distortion constants. This could also be used to compare to theory to help with the structure determination, but was not presented.

(Reply) The centrifugal distortion constants could add some additional spectroscopic evidence. Nonetheless, with the new current data set obtained at low J values, only a rigid rotor analysis was possible according to the expected rigidity of both axial and equatorial forms.

^{14}N nuclear quadrupole coupling constants are the most precise way to discriminate between axial and equatorial forms. The unambiguous conformational identification based on the analysis of the ^{14}N hyperfine structure have been carried out in many biomolecular systems:

- *Alonso J. L., López J. C. Microwave Spectroscopy of Biomolecular Building Blocks. In: Gas-Phase IR Spectroscopy and Structure of Biological Molecules. 2015. p. 335–401.*

- *Cabezas C., León I., Alonso E. R., Alonso JL. The Shape of Dipeptides: Insights from Rotational Spectroscopy. In: Thygesen TL, editor. Dipeptides and Tripeptides: Advances in Applications and Research. Nova Science Publishers; 2020.*
- *Cabezas C., León I., Alonso E. R., Alonso J. L. The Shape of Dipeptides: Insights from Rotational Spectroscopy. In: Thygesen TL, editor. Dipeptides and Tripeptides: Advances in Applications and Research. Nova Science Publishers; 2020.*
- *Alonso E. R., León I., Alonso J. L. The role of the intramolecular interactions in the structural behavior of biomolecules: Insights from rotational spectroscopy. In: Intra- and Intermolecular Interactions Between Non-covalently Bonded Species. Elsevier; 2021. p. 93–141. (Developments in Physical & Theoretical Chemistry).*
- *Alonso J. L., Vaquero V., Peña I., López J. C., Mata S., Caminati W. All five forms of cytosine revealed in the gas phase. Angew Chemie - Int Ed. 2013;52(8):2331–4.*
- *Peña I., Cabezas C., Alonso J. L. The nucleoside uridine isolated in the gas phase. Angew Chem Int. Ed. Engl. 2015 Mar 2;54(10):2991–4.*
- *Léon I., Tasinato N., Spada L., Alonso E. R., Mata S., Balbi A. et al. Looking for the Elusive Imine Tautomer of Creatinine: Different States of Aggregation Studied by Quantum Chemistry and Molecular Spectroscopy. Chempluschem. 2021 Oct 1;86(10):1374–86.*

We have added the following sentence in the main text (page 10) to reinforce this point further:

"Thus, the experimental values of the χ_{aa} and χ_{cc} diagonal elements of the ^{14}NI nuclear quadrupole coupling tensor vary from -1.023(14) to -4.632(49) and from 1.583(19) to 2.064(89), respectively, in excellent agreement with the predicted values shown in Table 1."

Finally, the conformational identification is further corroborated by dipole moment selection rules. Thus, the main difference between rotamers I and II is the absence of c-type lines for rotamer II, which is consistent with the low predicted value of the μ_c dipole moment component (0.6 D) for the equatorial conformer.

4. It is unclear to me that the method for creating the gas phase cytosine, laser ablation, would give a definitive answer as to the lower energy structure. Laser vaporization sources are known for providing a lot of energy to the system. The mechanistic pathway to the bottom of the well is then reliant on the expansion gas utilized and how it goes about cooling the resultant species. The authors already claim there are products due to photofragmentation, implying a lot of introduced energy, could they provide information on how the relaxation process takes place to ensure the relaxation to the global minimum?

(Reply) "The referee is right regarding that laser ablation vaporization techniques can provide much energy to the system, and several photofragmentation species were indeed found in the spectrum (see L. Kolesniková, I.León,E. R. Alonso, S. Mata and J.L. Alonso Angew. Chem. 2021, 133, 24666). The observed relative abundances of axial and equatorial conformers of cytisine in the supersonic expansion are the result of a series of processes that include the laser-vaporization of solid cytisine, the seeding of cytisine molecules in the region where the laser ablation plume and the carrier gas stream cross each other, and the collisional cooling occurring in the subsequent supersonic expansion. The relative population of the axial and equatorial conformers of cytisine would be brought close to that of thermodynamic equilibrium at the temperature of the carrier gas only if a high collision rate exists in the seeding region. In this case, the population of the different alanine conformers is brought to the equilibrium ratio in the pre-

expansion mixture, and thus the relative population of conformers in the jet can be directly related to the preexpansion equilibrium distribution.

Reviewer 2:

The authors investigated the cytisine by means of rotational spectroscopy, identifying the axial and equatorial conformer. Contrary to what one might think, the axial conformer is more stable and they managed to motivate the experimental evidence. The experimental work is certainly well developed, the results are widely supported by the data that are original and coherent. The computational part is also well described and amply supports the results obtained.

My concerns arise from the context developed within the article. The authors speak of Nicotinic Agonist Cytisine bringing as references some works including the famous: Sheridan, R. P .; Nilakantan, R .; Dixon, J. S .; Venkataraghavan, R. The Ensemble Approach to Distance Geometry: Application to the Nicotinic Pharmacophore. J. Med.Chem. 1986, 29 (6), 899–906. https://doi.org/10.1021/jm00156a005. However in that article they speak of a three-center pharmacophore while the authors continue to highlight only a two-center model (A - B). In this context the three-dimensionality is lost and it is not clear whether the molecule actually reflects the selected criteria.

(Reply) "As the reviewer points out, we initially based our results following the biochemical discussion reported in the work "Approach to Distance Geometry: Application to the Nicotinic Pharmacophore. (J. Med. Chem. 1986, 29 (6), 899–906) " in which authors consider a pharmacophore consisting on three centers to explain the biological activity of nicotine and its derivatives. However, "point C" corresponds to any atom bonded to the B center. In some cases, it does not even need to be an atom (see nicotine and strychnine in Chart I of the mentioned paper) where C's are "dummy" atoms. In this context, we explored and extended to a more recent bibliography (highlighting the work from Celie P. H. N. et al. Nicotine and carbamylcholine binding to nicotinic acetylcholine receptors as studied in AChBP crystal structures (Neuron. 2004;41(6):907–14. https://doi.org/10.1016/S0896-6273(04)00115-1). In this paper, the authors characterize the structure of nicotine binding to the receptor. From this structure, the authors conclude that the points required for the ligand to bind the receptor are both nitrogen atoms. For these reasons, we decided to consider only two centers as the contact points with the receptor.

Furthermore, the discussion also focuses on the subsequent protonation of the molecule. But what would be the difference between the axial and the equatorial form once protonated? The two conformers would be the same without any distinction.

(Reply) "The referee is right regarding that the discussion mainly focuses on the protonation of cytisine since this process is key to allowing the molecule to play its biological role. While the positive action of both protonated axial and equatorial forms is expected to be the same, the protonation process itself is more hampered for the equatorial form, as reported in our work. However, this process is more favorable for the axial conformation, which is the predominant structure unveiled in our experiment."

Then there are some typos that authors should take into consideration:

1) Page 5: I shoul be italics

2) Figure 2: I and F should be italics

3) Table 1: They wrote B3LYP. Is it right? Or they use an empirical dispersion?

4) Page 10: NI-H, write I in pedix

(Reply) "We thank the reviewer for pointing out these typos. We have revised and corrected the typographic errors."

Reviewer 3:

The paper reports microwave spectroscopic results of cytosine, an alkaloid with biological activity. Although the molecule is important for its biological activity, the size of the molecule is fairly large for detailed spectroscopic investigation. The authors group has been successfully obtained such molecules in the gas phase and characterized them by rotational spectroscopy. They were able to detect two conformers for this molecule determining relative abundances between them, based on quantum chemical calculations. This result is a key to discuss its biological activity. Thus, the paper contains unique new information not available by other methods, and worth publishing.

(Reply) "We thank the referee very much for the kind comments."

jz-2022-020214.R2

Name: Peer Review Information for "The Nicotinic Agonist Cytisine: The Role Of The NH···N Interaction"

Second Round of Reviewer Comments

Reviewer: 3

Comments to the Author

In spite of the comments by referee 1, reasoning of the assignment for the two conformers based on the dipole moment selection rule and the eQq constants of N_I is conclusive. Therefore, the paper has importance by confirming the existence of the two conformers with the predominance of the axial conformer. The paper is thus worth publishing as it is.

Reviewer: 1

Comments to the Author

All of my concerns have been adequately addressed and I suggest publication in JPCLett.

Author's Response to Peer Review Comments:

Professor Editor

Senior Editor, The Journal of Physical Chemistry Letters

J. Phys. Chem. Letters

September 14th, 2022

Dear prof. Editor,

Thank you again for considering our work (ID: jz-2022-020214.R1) entitled: "The Nicotinic Agonist Cytisine: The Role Of The NH···N Interaction" for publication in The Journal of Physical Chemistry Letters. A revised copy of our manuscript has been just uploaded including all the editorial issued that needed to be addressed. A point-by-point response detailing each formatting item may also be found below.

Best regards,

Prof. José Luis Alonso

(Author reply) We thank the editor for his comments. Following his suggestions, we have made the corresponding changes in the manuscript that are here indicated between quotations marks.

- Please make sure that the author affiliations between the manuscript file and the supporting-information file match exactly.

(Author reply) We have corrected the author affiliations.

- Title must match in three places: (1) manuscript file, (2) supporting information, and (3) ACS Paragon Plus.

(*Author reply) We have double-checked the title in manuscript file, in supporting information and in ACS Paragon Plus.*

- Author list must match in three places: (1) manuscript file, (2) supporting information, and (3) ACS Paragon Plus (Raul Aguado Vesperinas vs. Raul Aguado).

(*Author reply) We have corrected the author list.*

- Please number SI pages in the following format: "S1, S2..."

(*Author reply) We have modified the labelling of both Tables S1 and S3 in the SI.*

(Author reply) We, also, thank very much reviewers 1 and 3 for the kind comments.
